# Identification of Novel Feline Paramyxoviruses in Guignas (*Leopardus guigna*) from Chile

**DOI:** 10.3390/v12121397

**Published:** 2020-12-06

**Authors:** Michael Sieg, Irene Sacristán, Johannes Busch, Karen A. Terio, Javier Cabello, Ezequiel Hidalgo-Hermoso, Javier Millán, Denny Böttcher, Kristin Heenemann, Thomas W. Vahlenkamp, Constanza Napolitano

**Affiliations:** 1 Institute of Virology, Faculty of Veterinary Medicine, Leipzig University, An den Tierkliniken 29, 04103 Leipzig, Germany; Johannes.Busch@vetmed.uni-leipzig.de (J.B.); Kristin.Heenemann@vetmed.uni-leipzig.de (K.H.); Vahlenkamp@vetmed.uni-leipzig.de (T.W.V.); 2 Facultad de Ciencias de la Vida, Universidad Andres Bello, República 440, Santiago 7550196, Chile; isacristan.vet@gmail.com (I.S.); syngamustrachea@hotmail.com (J.M.); 3 Zoological Pathology Program, University of Illinois, 3300 Golf Road, Brookfield, IL 60513, USA; kterio@illinois.edu; 4 Facultad de Medicina Veterinaria, Universidad San Sebastián, Puerto Montt 5840000, Chile; javier.cabello@uss.cl; 5 Parque Zoológico Buin Zoo, Buin 9500000, Chile; ezequielhidalgovet@yahoo.com; 6 Instituto Agroalimentario de Aragón-IA2, University of Zaragoza-CITA,c/ Miguel Servet, 177, 50013 Zaragoza, Spain; 7 Fundación ARAID, Avda. de Ranillas, 50018 Zaragoza, Spain; 8 Institute of Veterinary Pathology, Faculty of Veterinary Medicine, Leipzig University, An den Tierkliniken 33, 04103 Leipzig, Germany; denny.boettcher@vetmed.uni-leipzig.de; 9 Departamento de Ciencias Biológicas y Biodiversidad, Universidad de Los Lagos, Avenida Fuchslocher 1305, Osorno 5290000, Chile; 10 Instituto de Ecología y Biodiversidad (IEB), Universidad de Chile, Las Palmeras 3425, Santiago 7800003, Chile

**Keywords:** guigna, feline, morbillivirus, paramyxovirus, phylogeny, kidney

## Abstract

The family of paramyxoviruses has received growing attention as several new species have been identified recently, notably two different clusters in domestic cats, designated as feline morbillivirus (FeMV) and feline paramyxovirus (FPaV). Their phylogenetic origin and whether wild felids also harbor these viruses are currently unknown. Kidney samples from 35 guignas (*Leopardus guigna*), a wild felid from Chile, were investigated for paramyxoviruses using consensus-RT-PCR. In addition, thirteen serum samples of guignas were screened for the presence of FeMV-specific antibodies by an immunofluorescence assay (IFA). Viral RNA was detected in 31% of the kidney samples. Phylogenetic analyses revealed two well-supported clusters, related to isolates from domestic cats, rodents and bats. No significant histopathology changes were recorded in infected guignas. Serology identified two samples which were positive for FeMV-specific antibodies. Our study highlights the diversity of paramyxovirus infections in felids with special emphasis on guignas from Chile.

## 1. Introduction

The guigna (*Leopardus guigna*), one of the smallest felids on earth [1], along with Geoffroy’s cat (*L. geoffroyi*), belongs to the genus Leopardus within the Ocelot Lineage that diverged from a common feline ancestor approximately 2.8 million years ago [2]. Based on morphological data, guignas are further divided into two subspecies, *L. g. tigrillo* (northern subspecies) and *L. g. guigna* (southern subspecies), living in separated geographical regions with different ecological landscapes and climates of Chile and Argentina [3]. Guignas require vegetation cover, and thus mainly use areas of Mediterranean woodlands and temperate rainforests. Due to their restricted distribution to some parts of Chile (30°–48° S) and a small region of south-western Argentina (39°–46° S west of 70° W), habitat loss, landscape fragmentation and human persecution have been causing a decline of this species within the last decades. Climate change, deforestation and human–felid conflicts are the most important issues impacting conservation of the guigna population [4]. Therefore, guignas are classified as Vulnerable on the International Union for Conservation of Nature (IUCN) Red List and are one of the most-threatened South American cat species [5]. Infectious agents such as feline immunodeficiency virus (FIV) and feline leukemia virus (FeLV) [6], as well as canine protoparvovirus [7], can worsen the current situation for guignas as these agents affect domestic and wild felids, causing significant morbidity or even death [8]. Another carnivore virus, canine morbillivirus virus (CDV), can have even more devastating effects in wild felids, as shown by an epidemic in Serengeti lions (*Panthera leo*), accounting for the death of approximately one-third of the whole population [9]. High mortalities were also found in Amur tigers [10]. Exposure to CDV has been documented in many other wild felids [11], including members of the genus *Lynx*, Namibian cheetahs (*Acinonyx jubatus*) and caracals (*Caracal caracal*), pumas (*Puma concolor*) and the Argentinian Geoffroy’s cats (*Leopardus geoffroyi*). However, most of these examples reported serological evidence of exposure and were not associated with disease, raising the question whether CDV infections in non-canid species should be reconsidered as normal rather than incidental hosts of this virus [12].

CDV belongs to the family of *paramyxoviridae*, composed of enveloped, single-stranded RNA viruses affecting a broad range of wild free-ranging and domestic animals [13]. In 2012, a new paramyxovirus was discovered in domestic cats from Hong Kong, and received the name feline morbillivirus (FeMV-1, formerly known as FmoPV) [14]. Initially, the virus was described to be associated with tubulointerstitial nephritis but subsequently, detection of the virus in Europe [14,15,16,17], Asia [18,19,20,21] and the Americas [22,23] showed that the situation is more complex, as some authors found a connection to urinary tract disease, while others did not. Furthermore, a second genotype of FeMV, now designated as FeMV-2, was detected in domestic cats from Germany. Strains of this genotype showed only 71% homology to FeMV-1 based on the nucleotide level of whole genome comparisons [24]. In addition, FeMV-unrelated paramyxoviruses were detected in domestic cats from Germany [15], the UK [17] and Japan [25], designated as feline paramyxoviruses (FPaV). All of these descriptions point towards the complexity of the family *paramyxoviridae* in cats. Our aim was to investigate the presence and diversity of FeMV in a wild felid, for which no survey has been reported to date.

## 2. Materials and Methods 

### 2.1. Sample Collection, RNA-Isolation, PCR Amplification and Histopathology

We screened cryopreserved (at −20 °C) kidney samples from road-killed guignas and guignas that arrived at wildlife rescue centers for various reasons (dog attacks, human persecution and fire injures) and died after admission, collected between 2008 and 2018 in Chile. Samples originated from Chiloe Island and the Chilean continent. RNA from kidneys was isolated using the RNeasy Mini Kit (Qiagen, Hilden, Germany) following the manufacturer’s instructions. Consensus one-step-nested-RT-PCR capable of amplifying all members of the *paramyxoviridae* family was applied exactly as described previously [26] using the ‘SuperScript III One-Step RT-PCR System with Platinum Taq High Fidelity’ system (Thermo Fisher Scientific, Waltham, MA, United States of America). We used the primer pairs PAR-R and PAR-F1/F2 as wells as RES-MOR-HEN-R and RES-MOR-HEN-F1/F2 targeting two different conserved nucleotide regions of the viral polymerase (RdRp, L gene), resulting in PCR fragments of approximately 610 and 495 bp, respectively. Both primer pairs were used for screening purposes as they differ in specificity and sensitivity [26].

For histopathology, replicate kidney samples were preserved in 10% neutral buffered formalin, processed routinely for hematoxylin-eosin staining, and were evaluated by a veterinary pathologist blinded to molecular data. Sections were investigated for glomerular (sclerosis and mesangial expansion), tubular (necrosis, atrophy, expansion and casts) and interstitial (inflammation and fibrosis) changes using previously described criteria [27].

The collection of samples was done under considerations of animal welfare and ethical aspects with the approval of Animal Ethics Committee of the Institute of Ecology and Biodiversity in Universidad de Chile, resolution of 20 November 2015. Frozen tissues were imported to Germany under the permission number 24-9152.81 (EFG-No.: 55/2018) provided by the ‘Saxon State Ministry for Social and Consumer Protection’, Dresden, Germany. In addition, samples were imported in accordance with the Convention on International Trade in Endangered Species of Wild Fauna and Flora (CITES), certificate no.: DE-E-03269/18, German Federal Agency for Nature Conservation, Bonn, Germany.

### 2.2. Phylogenetic Analysis

For phylogenetic characterization, PCR fragments were purified using a Gel/PCR DNA Fragments Extraction Kit (Geneaid, Taiwan) followed by sequencing via the Sanger’s dideoxy termination method by a commercial company (Microsynth Seqlab, Göttingen, Germany). Phylogeny was based on sequences (409 bp) derived from the RES-MOR-HEN-primers. Chromatogram files were analyzed with BioEdit software and edited sequences were screened at the NCBI website using the Basic Local Alignment Search Tool (BLAST, https://www.ncbi.nlm.nih.gov/blast). Phylogenetic analyses were conducted by calculating genetic distances employing the general time reversible model with gamma distributed invariant sites (GTR + I) at the nucleotide level using the MEGA-X software. A phylogenetic tree was built by the maximum likelihood method with 1000 bootstrap replicates [28].

### 2.3. Serological Analysis

Serum samples were investigated by using a previously established immunofluorescence assay (IFA) for both genotypes, FeMV-1 and FeMV-2 [29]. In brief, CrFK and LLC-KM2 cells were infected at a low multiple of infection (MOI) of 0.01 with FeMV-1 (accession no. MG563820.1) and FeMV-2 (accession no. MK182089.1) strains, respectively. Viruses were previously isolated from urine samples of two persistently infected cats from Germany [24,30]. Five days after infection, cells were fixed with 80% acetone, incubated with 5% (*w*/*v*) bovine serum albumin (BSA) in phosphate buffered saline (PBS) for 30 min at 37 °C to block unspecific binding sites. Cat sera were diluted 1/100 (*v*/*v*) in PBS, applied to the fixed and blocked cells and were incubated overnight at 4 °C. Unbound antibodies were removed by repeated washing with PBS and specific interactions were visualized using a 1/500 diluted goat anti-cat IgG (H+L) Alexa Fluor 488 conjugated antibody (Dianova, Hamburg, Germany). Mock-infected cells served as negative controls for each sample. Antibodies against CDV were detected by the use of virus neutralization assay (VNT) as described previously [31].

### 2.4. Statistical Analysis

QuickCalcs website (https://www.graphpad.com/quickcalcs/contingency2/) was used to determine significance between PCR positive and negative guignas and histologic lesions by the two-tailed fishers exact t-test.

### 2.5. Virus Isolation Attempts

Kidney samples were homogenized (TissueLyser II, Qiagen, Hilden, Germany) in PBS. Homogenates were clarified by low-speed centrifugation (500× *g*, 10 min), supernatants were diluted 1:2 with Dulbecco’s Modified Eagle’s Medium (DMEM) and applied to sub-confluent Vero (CCL-81), LLC-MK2 and CrFK cells. After two hours, the inoculum was replaced with Dulbecco’s Modified Eagle’s Medium (DMEM) including 2% heat-inactivated fetal bovine serum (FBS). Cells were grown in a humidified atmosphere with 5% CO_2_ for seven days with daily inspection for the presence of CPE. Three blind passages were performed until cell culture supernatants were tested for paramyxoviruses via consensus-RT-PCR.

## 3. Results

**Figure 1:**
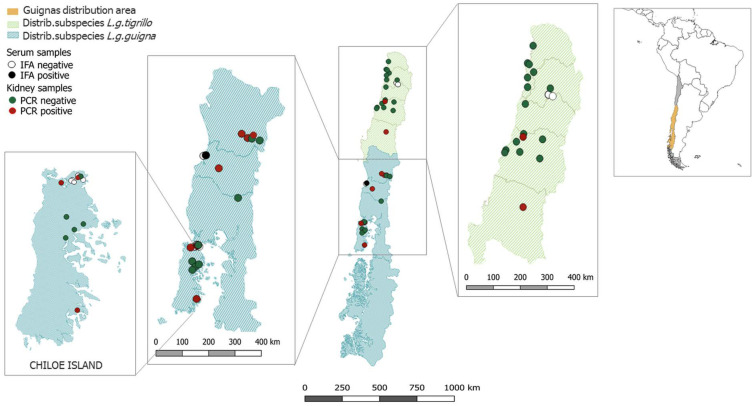
Sampling area of guignas used for PCR and serological analysis. Individual kidney samples of guignas are shown as green (PCR-negative) or red (PCR-positive), whereas serum samples are shown as white (IFA-negative) or black (IFA-positive) circles.

Kidneys of 35 animals (30 adults, 4 juveniles and 1 of non-determined age) composed of 19 female and 16 male guignas were subjected to PCR analysis. Eleven samples (31.4%) were found to be paramyxovirus-positive by using the more sensitive primer pair RES-MOR-HEN-R and RES-MOR-HEN-F1/F2. Positive animals originated from the central-south region of Chile, in which 1/15 of the southern subspecies (*L. guigna tigrillo*) and 9/18 of the northern subspecies (*L. guigna guigna*) were paramyxovirus PCR-positive (Figure 1). This difference in subspecies prevalence was found to be significant (Fisher’s *p* = 0.0094).

Two samples originated from animals of the transition zone between both subspecies in which one kidney sample was paramyxovirus PCR-positive. No significant differences in paramyxovirus prevalence between female (6/19) and male (5/16) animals were found (Fisher’s *p* = 1.0). Sequences have been deposited at GenBank, with the accession numbers MN850340–MN850350.

**Figure 2:**
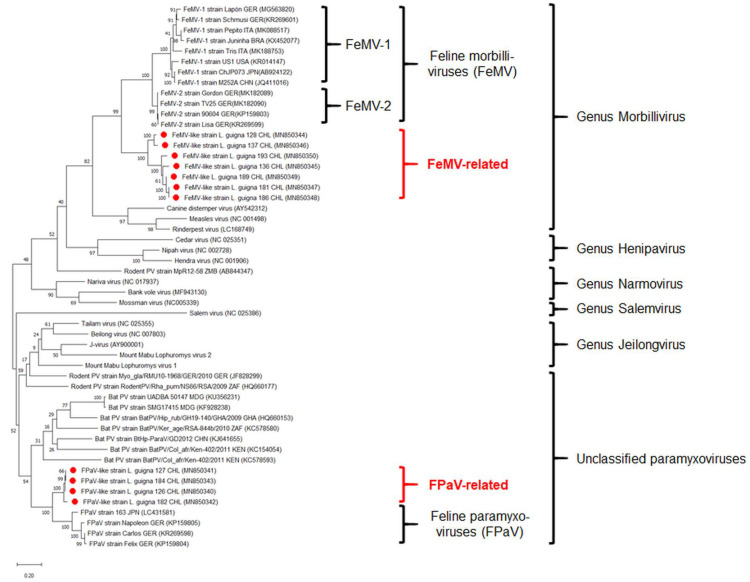
Maximum likelihood phylogenetic tree of paramyxoviruses including novel viruses detected in guignas from Chile (red dots). Accession numbers are shown in brackets. The tree is drawn to scale, with branch lengths measured in the number of substitutions per site.

Sequencing of the obtained PCR products revealed two different well-supported clades of paramyxoviruses: tentatively named ‘FeMV-related’ (7 of 35 animals, 20%) and ‘FPaV-related’ (4 of 35 animals, 11.4%) clusters (Figure 2). This diversity was also confirmed by PCR results using the primer pair PAR-R and PAR-F1/F2, although only eight samples were paramyxovirus-positive with this protocol (GenBank accession numbers MW027007–MW027014). The guigna FPaV-related sequences originated from four animals, three females and one male. Three samples were derived from animals from Chiloe Island (LG 126, LG 127 and LG 184), whereas the remaining FPaV-related positive animal (LG 182) lived on the Chilean continent. Genetic variability among sequences from Chiloe Island was found to be low with nucleotide homologies ranging between 99.26% and 99.75%. These sequences could be distinguished from the FPaV-related isolate of the Chilean continent having 95.56–95.80% nucleotide homology to the strains from the island. Highest identities to known paramyxoviral isolates were obtained with FPaV strains from domestic cats in Germany [15], the UK [17] and Japan [25], showing 80%, 81% and 82% homology, respectively (summarized in Table 1). In addition, these viruses were more distantly related to paramyxoviruses found in bats and rodents, with nucleotide homologies of 76% and 73%, respectively [32,33].

FeMV-related sequences were detected in seven guignas, three female and four male animals. Based on their geographic origin, viral sequences of guignas from Chiloe Island (LG 128 and LG 137) and isolates from the Chilean continent (LG 136, LG 181, LG186, LG 189 and LG 193) were not as homogeneous as in the FPaV-related cluster but instead showed only 83–85% nucleotide identity, forming two well-defined sub-clusters (Figure 2). Sequences were related to morbilliviruses found in domestic cats worldwide, with highest homology (74–75%) to FeMV isolates from Brazil [23] and China [14], respectively. Furthermore, FeMV-related viruses were related to paramyxoviruses found in rodents [34] and bats [35], having a nucleotide homology of about 70%. No co-infections of FPaV-related and FeMV-related viruses were detected in the investigated kidney samples. Virus isolation attempts were not successful.

Histopathological analysis of kidney samples from all investigated guignas exhibited only slight morphologic changes in glomerular architecture, e.g., faint to moderate cell proliferation and mesangial expansion. No significant differences were detected when comparing paramyxovirus-positive versus paramyxovirus-negative samples (Fisher’s *p* = 0.6871). Tubular variables (necrosis, atrophy, expansion and casts) were inconspicuous in all samples. Minimal inflammation and fibrosis of the interstitium were only seen in seven guignas respectively, but were unrelated to paramyxovirus PCR results.

**Figure 3:**
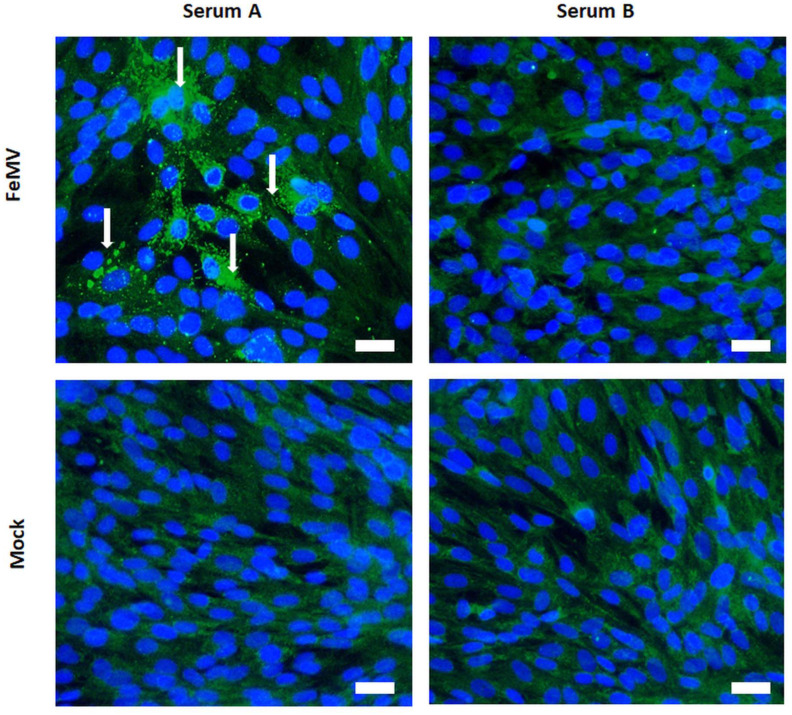
Serology of analyzed guigna serum samples from Chile. Representative result of a serological analysis via immunofluorescence assay. FeMV-specific antibodies were detected in a guigna serum sample (serum A) illustrated by fluorescence staining of perinuclear and cytoplasmic viral inclusion bodies (white arrows). In comparison, an IFA-negative sample (serum B) is shown on the right. Scale bar represents 20 µm.

For serological investigation, thirteen serum samples of guignas were collected in 2008 (*n* = 8) and in 2012 (*n* = 5) and analyzed for the presence of FeMV-specific antibodies via IFA. As a result, two samples (15.4%) were IFA-positive for both antibodies, FeMV-1 and FeMV-2 (Figure 3). No FeMV-1-only or FeMV-2-only positive serum samples were observed. All samples were negative for CDV antibodies by VNT.

## 4. Discussion

In the last decade, numerous new paramyxoviruses have been described with special emphasis on rodents and bats [32,33,34,35]. Most of these viruses were detected by consensus-nested-PCR [26] and sequencing of partial nucleotide sequences derived from conserved regions of the viral polymerase. In the current study, we applied this methodological approach to kidney samples (*n* = 35) from *Leopardus guigna* with an overall PCR-positive rate of approximately 31%. Differences in the detection rate of the two consensus primer sets used (RES-MOR-HEN vs. PAR primers) are not surprising as the latter (family-derived) have been described to have a broader reactivity but a ten-fold lower detection limit in comparison to genus-derived consensus primer pairs [26,36]. In our study, only kidney samples were examined, so it cannot be excluded that other organs may also be affected by these guigna paramyxoviruses. For the closely related feline morbilliviruses of domestic cats, histopathological data suggest that other organs (spleen, urinary bladder and immune cells) can be infected with FeMV-1 as well [37]. This finding was also supported by in vitro experiments showing that epithelial cells of the lung, alveolar macrophages and brain tissues are permissive for FeMV-2 under laboratory conditions [24,37]. On the other hand, in a recently detected infection of black leopards (*Panthera pardus*) with FeMV-1, only kidneys were affected [38]. Further studies addressing surveillance of paramyxoviruses in wildlife animals should take into account that organs other than the kidney may also be a target of viral replication and should therefore be sampled to elucidate the complete tissue tropism of these viruses.

The viral sequences from guignas could be divided into two distinct phylogenetic clusters resembling paramyxovirus diversity found in domestic cats [15,17,24,25]. FeMV-related and FPaV-related clades from guignas of the Chilean continent or Chiloe Island differed in their nucleotide sequences. This phenomenon may be explained by independent introductions of these viruses to the two guigna populations. Since animals from the mainland should not have any current direct contact with animals from the island (although there was some historical connection in the last glacial maximum) [4], it is possible that the observed sequence differences are the result of a co-evolution of the viruses with their respective hosts. On the other hand, it should be taken into account that domestics are indeed moved between the island and the continent so that cross-species transmission between wild felids and domestic cats could also explain the observed phenomenon. No differences in paramyxovirus prevalence between female and male animals were encountered, pointing toward a transmission route which is independent of the sex or sex-related behaviors. In contrast, significant differences in the proportion of paramyxovirus-positive animals were observed between the two guigna subspecies, in which *L. guigna guigna*, the southern subspecies, had significantly higher infection compared to *L. guigna tigrillo*. This is probably the result of differences between central and southern Chile in terms of bioclimatic conditions, animal densities, or other yet unknown reasons. From a biological point of view, there is no rationale for one guigna subspecies being more susceptible to paramyxoviruses than the other. No previous studies investigating several other pathogens in guignas revealed any evidence to predict differences between subspecies [6,39]. In addition, sample numbers of the current study were relatively low and therefore a statistical artefact cannot be completely excluded. Further large-scale investigations which consider a representative sampling size are needed to draw a final conclusion.

The detected guigna viruses were also related to paramyxovirus sequences found in kidneys of bats (*Hipposideros caffer*) from South Africa [32] and in urine and feces of *Microchiroptera* sp., *Myotis* sp. and *Scotophilus* sp. bats from Viet Nam and Cambodia [40]. Interestingly, rodents (e.g., *Rattus exulans*, *Rattus tanezumi* and *Lophuromys nudicaudus*) from Thailand, Myanmar and Malaysia harbor several similar paramyxoviruses but, in contrast, these viruses were detected in oral and rectal swabs of the animals [40]. The low nucleotide homology to viral sequences from rodents, bats and other known paramyxoviruses raises the question whether there are, yet unidentified, wild animals harboring related viruses which can explain viral evolution. Future surveillance programs including a broader pool of relevant animal species from Chile and neighboring countries as well as larger sample numbers are needed to address this issue.

The similarity of paramyxovirus diversity in guignas in comparison to domestic cats opens the discussion for historical cross-species transmission scenarios between domestic cats and wild felids. Transfer of infectious agents between domestic and wild cats is well-documented for several pathogens, such as FeLV and FIV [6]. In contrast to the herein described paramyxoviruses, FeLV and FIV sequences in guignas closely resembled known virus isolates from domestic cats. However, there are reports about FIV isolates from several wild felids such as lions [41] and pumas [42] which were clearly distinguishable from viruses found in domestic cats based on partial nucleotide sequence comparisons of the viral polymerase. In contrast, CDV strains from different domestic and wild carnivores show only limited sequence variability, mainly affecting the viral surface proteins as a consequence of receptor usage [43]. Since clades are well-separated between world-wide domestic cat and guigna viruses, past transmission events may have taken place by one or more interspecies virus jumps followed by a co-evolution within the guigna populations, leading to the currently observed phylogenetic diversity. However, Chilean domestic cats should be sampled and sequenced for paramyxoviruses, and then compared to guigna sequences to better assess this possible situation.

Confirmation of hypothetical transmission scenarios are hampered by the lack of whole genome sequences from paramyxoviruses of guignas, domestic cats, rodents and bats, as only short parts of whole viral genomes are available. On the other hand, the amplified sequence segments belong to a highly conserved region of paramyxoviruses which are widely accepted for phylogeny analyses and proposing new species [33]. In this study, attempts for virus isolation and uncovering further viral genome sequences were not successful, which may be explained by partial autolysis of the organs (the majority of the animals were road-killed) and prolonged storage (several years) at −20 °C, leading to RNA fragmentation or degradation and virus inactivation.

Although the initial description of FeMV was linked to kidney disease in domestic cats [11], no significant differences in histopathology of paramyxovirus-positive and paramyxovirus-negative guignas were seen. In contrast, FeMV-1 infections in black leopards (*Panthera pardus*) were associated with severe azotemia and tubulointerstitial nephritis [38]. Otherwise, several reports of FeMV infections in domestic cats could not find a distinctive connection to kidney diseases [17,20,37]. As the viruses detected in guignas clearly differ from FeMV and FPaV in domestic cats, continued monitoring and evaluation of complete necropsies are needed to fully ascertain whether these viruses are associated with disease.

Serological analysis of a limited number of serum samples from guignas revealed reactivity with FeMV-1 and FeMV-2. False-positive IFA results due to cross-reactive CDV antibodies were excluded by screening all serum samples via CDV-VNT [44], leading to the assumption that guignas may also be susceptible to FeMV strains of domestic cats. Recently, it was shown that domestic cats of Chile have a high seroprevalence for both FeMV genotypes [29]. As mentioned above, black leopards (*Panthera pardus*) were also shown to be susceptible to FeMV [38], which further support a hypothesis of possible FeMV infections in guignas. The seroprevalence of FeMV in guignas is in accordance with published results from studies of domestic cats from China [14], the UK [17] and Japan [45], although comparison with these investigations may be difficult due to differences in experimental techniques. In addition, it cannot be excluded that the observed IFA reactions are the result of cross-reactive FeMV-like antibodies, as whole genome sequences from these new strains are not yet available. Interestingly, seroprevalence of FeMV and observed prevalence of PCR-positive FeMV-related sequences in guignas are similar (15% vs. 20%, respectively), making it difficult to fully explore the molecular basis of the observed antibody prevalence without further information about antigen similarity of the respective viruses. Nevertheless, this is the first study documenting seroconversion of guignas against FeMV and/or FeMV-related strains.

## 5. Conclusions

We reported the identification of novel paramyxoviruses in guignas from Chile, forming two well-separated clades: FeMV-related and FPaV-related. Highest homologies were found to virus strains of domestic cats and more distantly related viruses of rodents and bats. Furthermore, we report supporting histopathological and serological data which points to the possibility of guigna infections with FeMV strains circulating in domestic cats. The impact of these viruses to the population persistence and health status of guignas is currently unknown and needs to be further investigated for this threatened wild felid.

## Figures and Tables

**Table 1:** Characteristics of paramyxovirus PCR-positive guigna samples.

Identity ^1^	Animals ^1^ (Accession Number)	Subspecies ^1^	Origin	Highest Nucleotide Homology to (Accession No.)	Relatedness to Other Paramyxoviruses
FPaV- related	LG 126 (MN850340)	LGG	Chiloe Island	83% FPaV *Felis catus*, Japan (LC431581.1)	76% bat paramyxovirus (KC578587.1) 74% Tenrec paramyxovirus (KF246040.1) 73% rodent paramyxovirus (JF828299.1)
	LG 127 (MN850341)	LGG			
	LG 184 (MN850343)	LGG			
	LG 182 (MN850342)	LGT	Chilean continent	82% FPaV (LC431581.1)	
FeMV- related	LG 128 (MN850344)	LGG	Chiloe Island	75% FeMV *Felis catus*, Brazil (KX452077.1)	70% bat paramyxovirus (MN602070.1)
	LG 137 (MN850346)	LGG			
	LG 136 (MN850345)	LGT	Chilean continent	74% FeMV *Felis catus*, China (JQ411016.1)	70% rodent paramyxovirus (AB844347.1) 70% bat paramyxovirus (MH259211.1)
	LG 181 (MN850347)	LGG			
	LG 186 (MN850348)	LGG			
	LG 189 (MN850349)	LGG			
	LG 193 (MN850350)	LGG			

^1^ LGG = *Leopardus guigna guigna*; LGT = *Leopardus guigna tigrillo*; LG = *Leopardus guigna*; FPaV = feline paramyxovirus; FeMV = feline morbillivirus.
